# Endothelial Antioxidant-1: a Key Mediator of Copper-dependent Wound Healing *in vivo*

**DOI:** 10.1038/srep33783

**Published:** 2016-09-26

**Authors:** Archita Das, Varadarajan Sudhahar, Gin-Fu Chen, Ha Won Kim, Seock-Won Youn, Lydia Finney, Stefan Vogt, Jay Yang, Junghun Kweon, Bayasgalan Surenkhuu, Masuko Ushio-Fukai, Tohru Fukai

**Affiliations:** 1 Departments of Medicine (Section of Cardiology) and Pharmacology, Center for Cardiovascular Research, Center for Lung and Vascular Biology, University of Illinois at Chicago, Chicago IL60612, USA; 2 Jesse Brown Veterans Affairs Medical Center, IL60612, Chicago, USA; 3 Department of Pharmacology, Center for Lung and Vascular Biology, Center for Cardiovascular Research, University of Illinois at Chicago, Chicago IL60612, USA; 4 X-ray Science Division, Argonne National Laboratory, Argonne, IL60439, USA; 5 Department of Anesthesiology, University of Wisconsin, Madison, WI 53726, USA.

## Abstract

Copper (Cu), an essential nutrient, promotes wound healing, however, target of Cu action and underlying mechanisms remain elusive. Cu chaperone Antioxidant-1 (Atox1) in the cytosol supplies Cu to the secretory enzymes such as lysyl oxidase (LOX), while Atox1 in the nucleus functions as a Cu-dependent transcription factor. Using mouse cutaneous wound healing model, here we show that Cu content (by X-ray Fluorescence Microscopy) and nuclear Atox1 are increased after wounding, and that wound healing with and without Cu treatment is impaired in Atox1^−/−^ mice. Endothelial cell (EC)-specific Atox1^−/−^ mice and gene transfer of nuclear-target Atox1 in Atox1^−/−^ mice reveal that Atox1 in ECs as well as transcription factor function of Atox1 are required for wound healing. Mechanistically, Atox1^−/−^ mice show reduced Atox1 target proteins such as p47phox NADPH oxidase and cyclin D1 as well as extracellular matrix Cu enzyme LOX activity in wound tissues. This in turn results in reducing O_2_^−^ production in ECs, NFkB activity, cell proliferation and collagen formation, thereby inhibiting angiogenesis, macrophage recruitment and extracellular matrix maturation. Our findings suggest that Cu-dependent transcription factor/Cu chaperone Atox1 in ECs plays an important role to sense Cu to accelerate wound angiogenesis and healing.

Copper (Cu), an essential nutrient and catalytic cofactor, plays important role in physiological process including angiogenesis which is required for reparative neovascularization and wound healing in response to injury^1^,^2^,^3^,^4^,^5^,^6^,^7^. Cu directly stimulates cell proliferation and migration in cultured endothelial cells (ECs)^1^. *In vivo*, Cu induces neovascularization and its concentration is increased in angiogenic tissue, while Cu chelators, which have been developed to treat Wilson disease (a disease of Cu toxicity), inhibit tumor growth and angiogenic responses^1^,^2^,^3^,^4^. Of importance, clinical trials for the treatment of solid tumors by Cu chelation show efficacy in disease stabilization^8^.

The earliest recorded application of Cu in wounds can be traced to Egyptian Papyrus written between 2600 and 2200 BC^9^,^10^. Indeed, Cu-induced accelerated wound healing has been demonstrated in various clinical and experimental settings^5^,^6^,^7^, such as severe burn trauma in children^11^, phosphorus burns^12^, and diabetic ulcers^13^. Wound healing proceeds in three overlapping but functionally distinct phases initiating with an inflammatory phase marked by infiltration of macrophages and secreted growth factors, followed by cell proliferation phase that includes angiogenesis, and then maturation phase that brings about extracellular matrix (ECM) remodelling and resolution of the granulation tissue^14^,^15^. However, little information is available regarding mechanism by which exogenous or endogenous Cu promotes wound angiogenesis and inflammation required for tissue repair.

Due to its potential toxicity in excess, intracellular Cu concentration under physiological conditions is tightly-regulated by Cu transporter and chaperone proteins^16^,^17^. Antioxidant-1 (Atox1) is cytosolic Cu chaperone that obtains Cu through the Cu importer CTR1, and transports Cu to the Cu-transporting/exporting ATPase (ATP7A) to maintain the intercellular Cu homeostasis^16^,^17^. In addition, Atox1 delivers Cu through ATP7A to the secretory Cu-dependent enzymes such as extracellular superoxide dismutase (ecSOD)^18^ or lysyl oxidase (LOX) which plays an important role in tissue remodelling and wound healing by regulating the cross-linking of collagen^19^,^20^,^21^,^22^,^23^. Furthermore, we previously reported that Atox1 also functions as a Cu-dependent transcription factor for cyclin D1 to promote cell proliferation *in vitro*^24^ or for cytosolic NADPH oxidase organizer p47phox to promote reparative ROS production in endothelial cells (ECs) and inflammatory cell recruitment, which are required for neovascularization in hindlimb ischemia model^25^. However, the specific role of endothelial and nuclear Atox1 for wound healing and repair remains unknown.

We thus performed the present study to investigate *in vivo* role of Atox1 in wound healing using cutaneous wound healing model with Atox1^−/−^ and EC-specific Atox1^−/−^ mice as well as Atox1^−/−^ mice treated with gene transfer of nuclear-targeted Atox1. Our study should provide novel insights into the Cu-dependent transcription factor/chaperone Atox1 in ECs as a potential therapeutic target for promoting reparative wound angiogenesis and healing.

## Results

### Both Cu and Atox1 are required for wound healing

**Figure 1:**
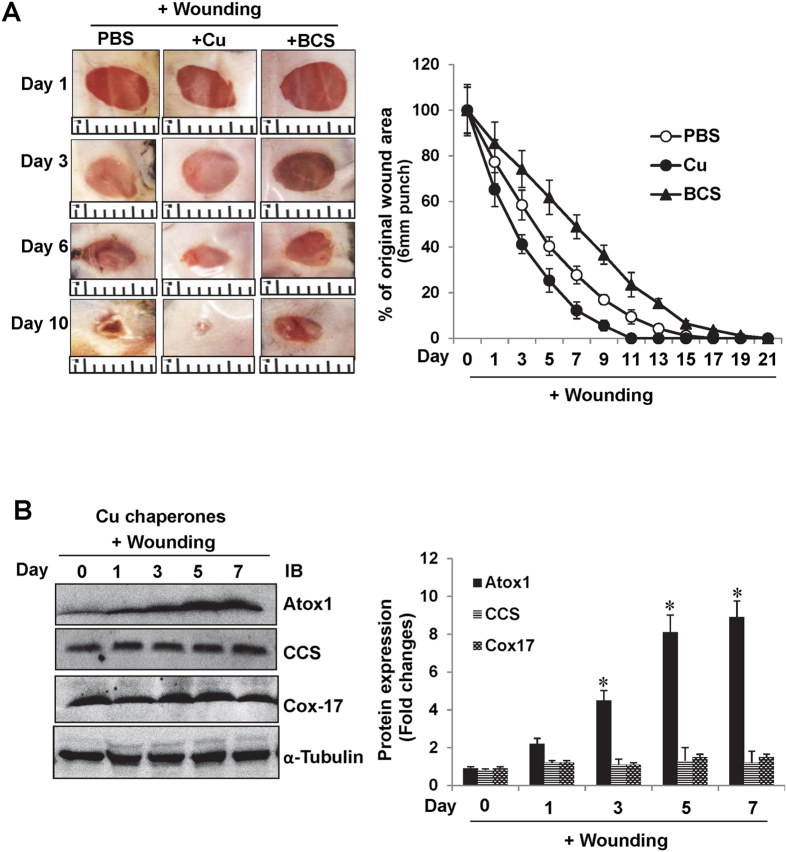
Exogenous and endogenous Cu promote wound healing. (**A**) Excisional cutaneous wounds were created using a 6 mm biopsy punch on the dorsal skin of WT mice, and treated with Cu (1.25 nmoles) or Cu chelator BCS (10 nmoles), or vehicle (PBS). Ruler notches, 1 mm. A graph represents the mean ± SE of wound closure rates (*p < 0.05; ^#^p < 0.05 vs PBS). (**B**) Expression of Cu chaperones, including Atox1, CCS, and Cox17, and α-tubulin (loading control) in wound tissues after injury. A graph represents mean ± SE at indicated date post-wounding (n = 3 at each time, *p < 0.001 vs. day 0).

**Figure 2:**
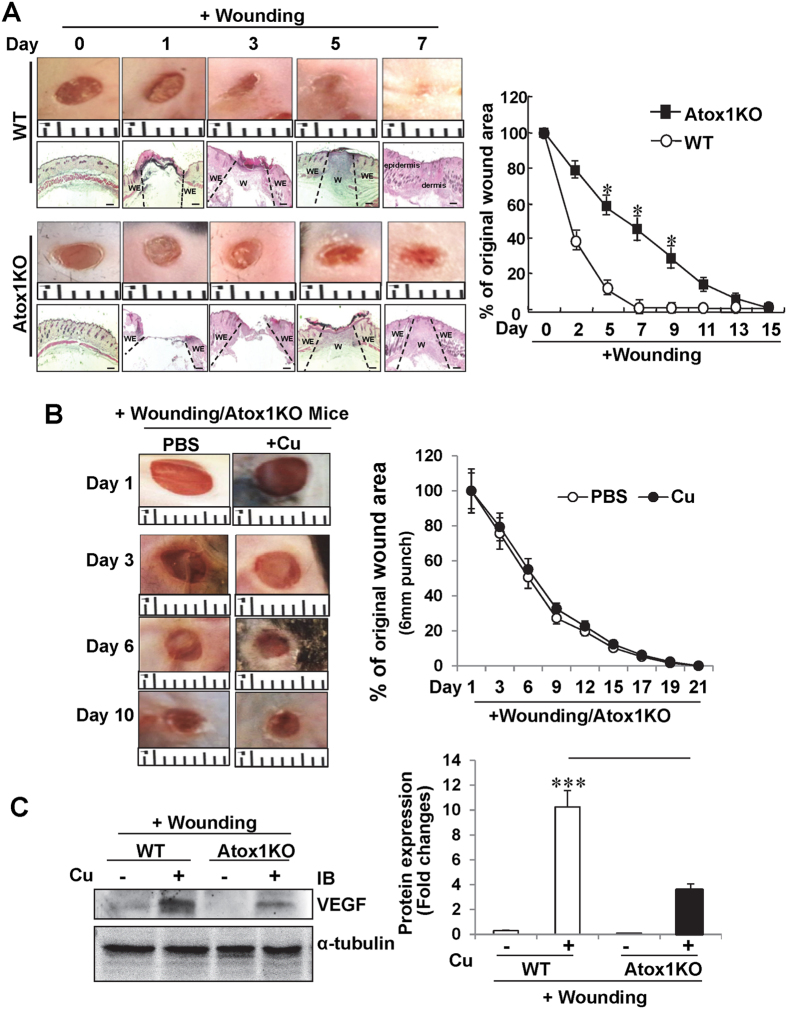
Atox1^−/−^ mice show impaired wound healing with and without Cu treatment. (**A**) Left panel, representative wound images and Hematoxylin & Eosin (HE) staining of cutaneous wounds created by 3 mm biopsy punch in WT and Atox1^−/−^ mice. A graph represents mean ± SE of wound closure rates (*p < 0.01 vs. WT, n > 6 per group). Ruler notches 1 mm, Scale bars = 100 μm. W: wound area; WE: wound edge (**B**) Cutaneous wounds created by 6 mm biopsy punch were treated with Cu (1.25 nmoles) or PBS in Atox1^−/−^ mice, as described in Fig. 1A. (**C**) Expression of VEGF protein and α-tubulin (loading control) in wound tissues treated with Cu or PBS at day 7 after injury in WT and Atox1^−/−^ mice (**p < 0.01 vs. WT; ***p < 0.001 vs. PBS-treated wound tissue).

Cu treatment accelerates wound closure and healing; however, underlying mechanism and role of endogenous Cu remains elusive^5^,^6^,^7^,^9^,^11^,^12^,^13^. To address this question, we made four excisional wounds on the dorsal skin in WT mice. Topical Cu treatment of the wound site significantly accelerated wound contraction and closures whereas Cu chelator BCS retarded the healing process (Fig. 1A). Figure 1B showed that wounding markedly increased expression of Cu chaperone Atox1, but not other chaperones such as CCS or Cox17 in a time-dependent manner. To determine the functional role of Atox1 in wound healing *in vivo*, we used Atox1^−/−^ mice and WT mice. Histological and quantitative morphometric analysis revealed that Atox1^−/−^ mice exhibited a marked decrease in wound closure rate as well as more rapid epithelialization by histological analysis (Fig. 2A). Figure 2B,C showed Cu-induced enhancement of wound healing as well as an increase in Atox1 (data not shown) and VEGF protein expression induced by wounding were markedly decreased in Atox1^−/−^ mice. These findings indicate that Atox1 senses Cu to promote wound healing at least via regulating VEGF expression, which has been shown to be essential for wound repair^26^.

**Figure 3:**
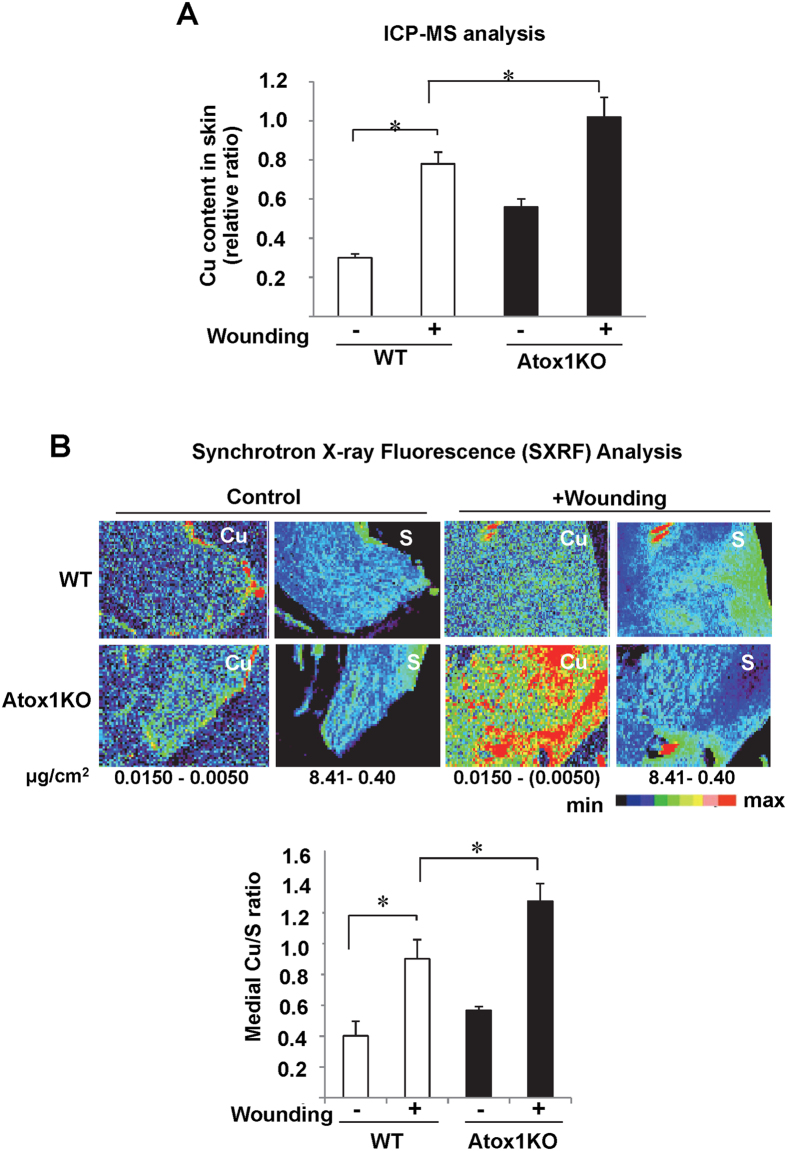
Abundance and spatial distribution of Cu in cutaneous wounds from Atox1^−/−^ and WT mice. Cu content in wound tissues at day 0 and 7 after injury in Atox1^−/−^ and WT mice was measured by inductively coupled plasma mass spectrometry (ICP-MS) (**A**) or synchrotron-based x-ray fluorescence (SXRF) (**B**). In (**B)**, SXRF scans (1–2 seconds per pixel) were performed in paraffin-embedded tissue (upper panel). The maximum and minimum threshold values in μg/cm^2^ are shown below each frame. Map of Cu shows areas of the lowest to the highest content scaled to a rainbow color (bottom). Total sulfur is used as a surrogate for total cellular protein and to visualize the morphology of tissue sections^27^,^28^. A graph represents mean ± SE of Cu content in the wounds (**A**) or medial Cu/S ratio (**B**). * < 0.05 vs. day 0 or WT.

To determine if endogenous Cu level is altered after wound injury, we measured the Cu content in wound tissues of WT and Atox1^−/−^ mice using ICP-MS (Fig. 3A) and SXFM (Fig. 3B). We found that Cu content was markedly increased in all the layers of wounded tissue with more in epidermis area at day 7 in WT mice. Of note, Cu levels were normalized by sulfur levels, which was used as a surrogate for total cellular protein and to visualize the morphology of tissue sections^28^,^28^. As shown in Fig. 3B, Atox1^−/−^ mice exhibit increased Cu levels in the non-wounded and wounded tissue as compared to WT mice. These results suggest that delayed wound healing in Atox1^−/−^ mice is not due to decreased Cu level in wounds but to the loss of Cu sensor function of Atox1.

### Atox1 facilitates Angiogenesis and inflammatory cell recruitment during wound healing

**Figure 4:**
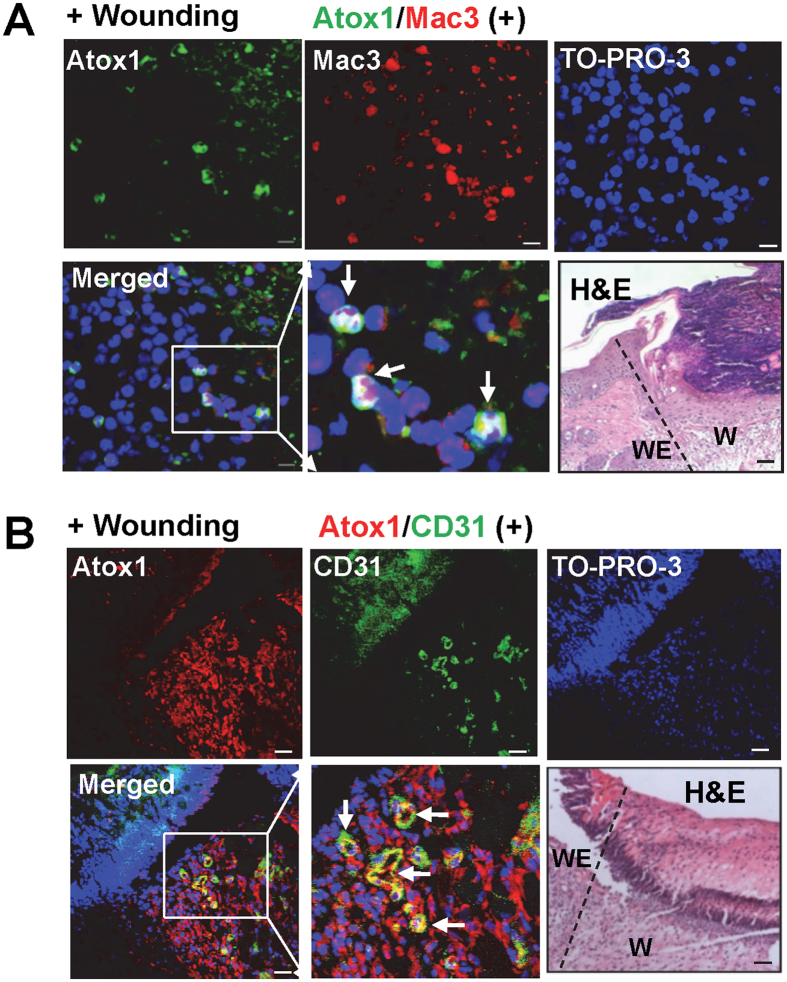
Atox1 is expressed in endothelial and inflammatory cells in the dermis during wound healing. (**A,B**) Hematoxylin & Eosin (H&E) and representative immunostaining for Atox1 (green), Mac3 (red, macrophage marker), TO-PRO3 (blue, nuclear marker) or their colocalization (Merge) on day 5 (**A**); and Atox1 (red), CD31 (green, EC marker), TO-PRO3 (blue, nuclear marker) or their colocalization (Merge) on day 7 (**B**) in the dermal region of wound tissues (W). Serial section stained with H&E (**A**,**B**), W: wound area; WE: wound edge, Scale bars = 10 μm.

**Figure 5:**
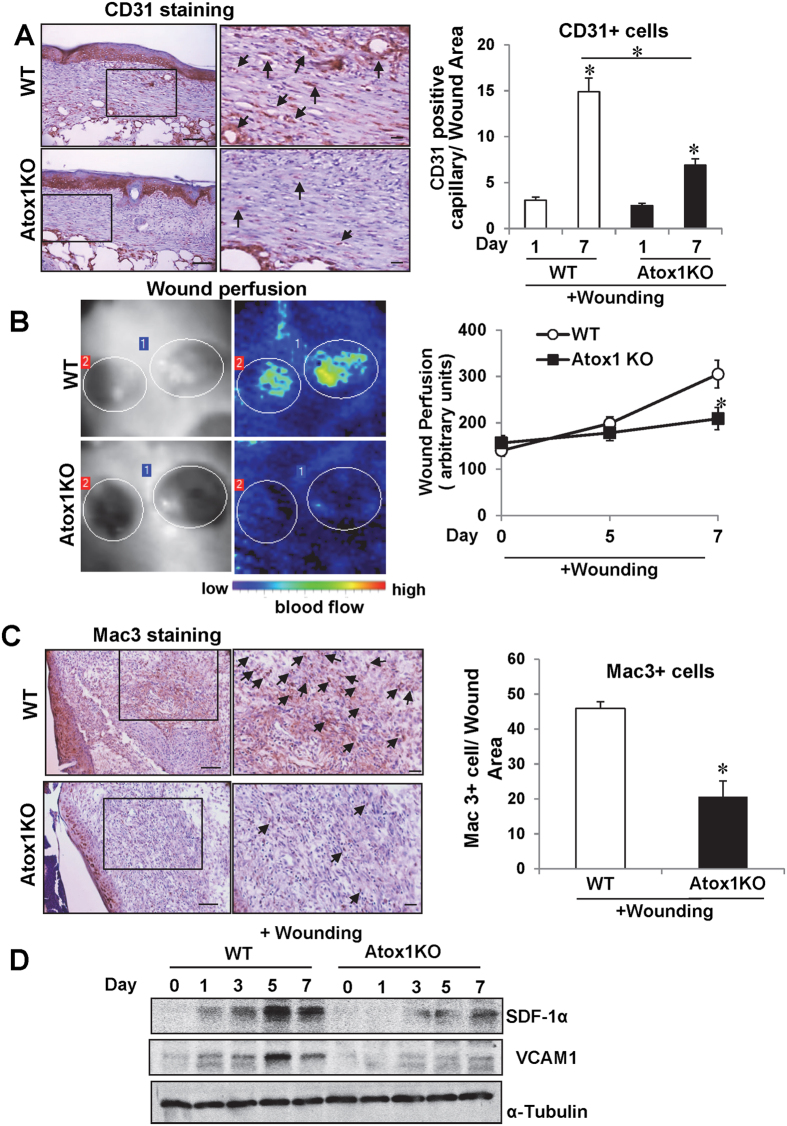
Atox1 facilitates angiogenesis and inflammatory cell recruitment during wound healing. (**A**,**C**) Representative images for CD31 (EC marker) at day 7, (**A**) and Mac3 (macrophage marker) at day 5 (**C**) in wound tissue of WT and Atox1^−/−^ mice. Boxed regions are shown at higher magnification in the right. Scale bar = 50 μm (left images); scale bar = 10 μm (right images). Graphs represent the mean ± SE of CD31^+^ and Mac3^+^ cells/wound area (n = 4). *p < 0.05 vs. day1 or WT. (**B**) Blood flow of wounds (wound perfusion) measured by laser Doppler. Representative images for blood flow (left) and quantitative analysis (right) in wound tissue are shown. A graph represents mean ± SE (n = 3) of wound perfusion. *p < 0.05 vs WT. (**D**) Expression of SDF-1α protein, VCAM1 protein and α-tubulin (loading control) in WT and Atox1^−/−^ mice in wound tissues at indicated dates after injury.

To gain insight into the mechanisms by which Atox1 is involved in wound healing which is dependent on inflammation at early phase and angiogenesis at later phase^15^,^29^,^30^ we performed immunofluorescence analysis to examine the expression and localization of Atox1 protein in skin tissues after wounding. Atox1 was highly expressed in Mac3^+^ macrophage at day 5 (Fig. 4A) and in capillary-like CD31^+^ ECs at day 7 (Fig. 4B). Of note, some Atox1 positive cells in ECs and macrophages were found in the nucleus. We then examined the functional role of Atox1 in wound-induced angiogenesis and inflammatory cell recruitment, which are involved in wound healing. Immunohistological and laser Doppler flow analysis demonstrated that wounding-induced increase in the number of capillary-like CD31^+^ ECs (Fig. 5A) and blood flow perfusion (Fig. 5B) in wound tissues were significantly inhibited in Atox1^−/−^ mice. Moreover, Atox1^−/−^ mice showed decreased number of infiltrated Mac3^+^ macrophage in wounded tissues (Fig. 5C), which was associated with reduced expression of chemokine SDF1α and VCAM1 (Fig. 5D) which was shown to be secreted by macrophages to promote revascularization and tissue repair^31^,^32^,^33^. These results suggest that Atox1 is required for wound healing by regulating angiogenesis and inflammatory cell recruitment to the injured sites.

### Atox1 in ECs is required for wound healing

**Figure 6:**
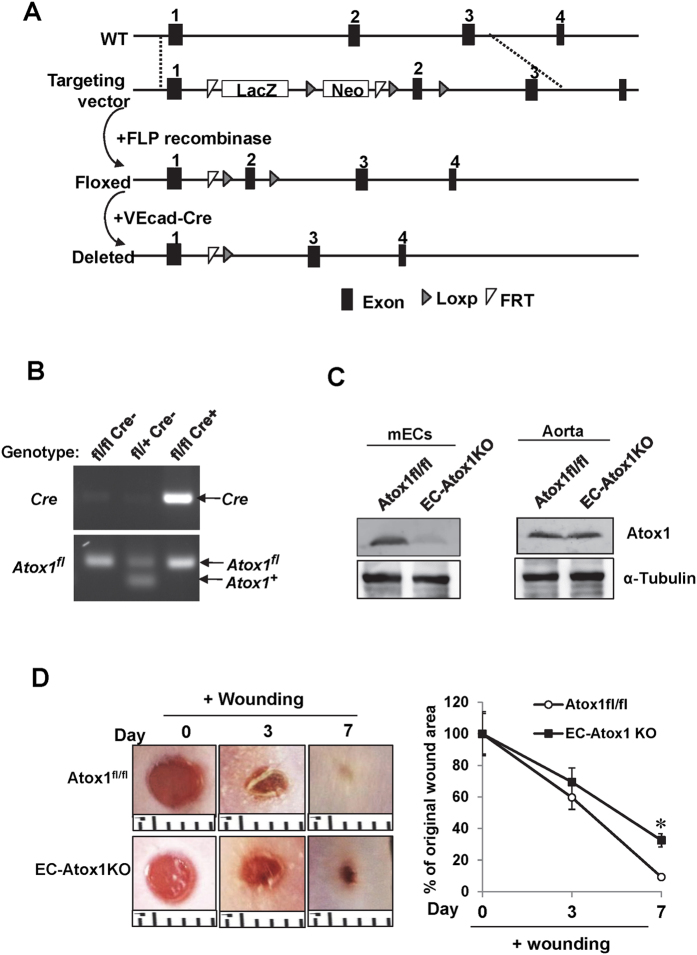
Endothelial cell (EC)-specific Atox1^−/−^ mice show delayed wound healing. (**A**) Strategy to generate EC-specific Atox1^−/−^ mice (EC-Atox1-KO) by crossing Atox1^fl/fl^ with constitutive VE-Cadherin (VEcad)-Cre deleter mice. Exon1-3 of the wild type and recombinant Atox1 locus are shown. The targeting construct included LoxP sites (grey triangles) that flanked exon 2 and a Neo gene cassette flanked by FRT sites (white triangles). The neo gene cassette was removed by crossbreeding with the FLP deleter strain expressing FLP1 recombinase to generate mice with desired floxed allele (Atox1^fl^). The exon 2-deleted Atox1 allele (Deleted) is depicted following excision by VEcad-Cre recombinase. (**B**) PCR analysis of genomic DNA isolated from mouse tails. Genotypes for Atox1^fl^, Atox1^+^, or VEcad Cre are indicated. (**C**) Atox1 and α-tubulin (control) protein expression in ECs isolated from mouse hearts (mECs) and aortas (which mainly consists of vascular smooth muscle cells) isolated from Atox1^fl/fl^ (control) and EC-Atox1 KO mice. (**D**) Representative image of cutaneous wounds (3 mm in diameter) (left panel) and a graph representing mean ± SE of the wound closure rate (right panel) of Atox1^fl/fl^ (control) and EC-Atox1 KO mice after wounding. (n > 3 per group, *p < 0.05 vs. control). Ruler notches 1 mm.

To demonstrate a specific role of endothelial Atox1 *in vivo*, we generated EC-specific Atox1 knockout mice (EC-Atox1 KO) by crossing Atox1^flox/flox^ mice with VE-cadherin Cre mice (Fig. 6A,B). ECs isolated from EC-Atox1^−/−^ mice demonstrated loss of Atox1 in ECs compared to those from control Atox1^flox/flox^ mice. By contrast, Atox1 expression in aorta, in which majority of cells are vascular smooth muscle, is not different in both mice (Fig. 6C). These results confirm the EC-specific reduction of Atox1 *in vivo*. Figure 6D showed that EC-Atox1 KO mice significantly delayed wound closure, suggesting that Atox1 in ECs plays an important role in wound healing.

### Nuclear Atox1 rescues the impaired wound healing in Atox1^−/−^ mice

**Figure 7:**
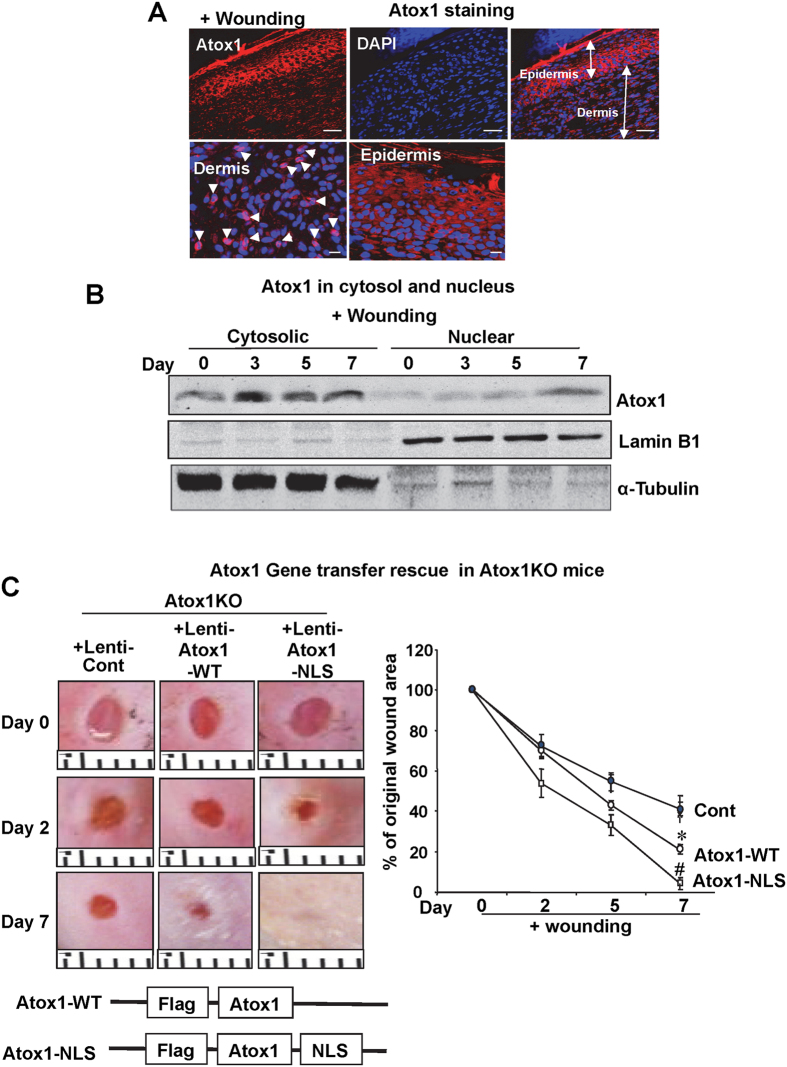
Gene transfer of nuclear-targeted Atox1 rescues impaired wound healing of Atox1^−/−^ mice. (**A**) Immunofluorescence showing Atox1 staining (red) in the nucleus of dermal region and in the cytosol of epidermal region and DAPI staining (blue, nuclear marker) at day 7 after wounding. Upper panel scale bars = 50 μm, lower panel scale bars = 10 μm (**B**) Nuclear and cytoplasmic fractions of wound tissue lysates were immunoblotted with anti-Atox1, α-tubulin (cytoplasmic marker), and laminin B1 (nuclear marker) antibody. (**C**) Lentiviruses expressing either control GFP vector virus or Atox1-WT or Atox1 with nuclear-target sequence (NLS) (Atox1-NLS) were injected to the cutaneous wounds of Atox1^−/−^ mice right after injury. Representative images (left) and a graph representing mean ± SE of wound closure rates. (n > 3 per group, ^#^p < 0.001; *p < 0.01 vs. control virus). Ruler notches 1 mm.

We previously reported that Atox1 functions not only as Cu chaperone but also as Cu-dependent transcription factor in ECs^25^. Indeed, immunofluorescence analysis showed that Atox1 was in the nucleus and the cytosol in the dermis area while in the cytosol in the epidermis area at day 7 (Fig. 7A). Consistent with this, subcellular fractionation assay in wounded skin lysates demonstrated that Atox1 expression in nuclear fraction was markedly increased at day 7 after wounding (Fig. 7B). To demonstrate the functional role of nuclear Atox1 in wound healing, we performed lentivirus-mediated gene transfer of nuclear targeted Atox1 (Atox1-NLS)^24^,^34^ in wound sites from Atox1^−/−^ mice. Impaired wound healing in Atox1^−/−^ mice was significantly rescued by Atox1-NLS and Atox1-WT (Fig. 7C). Of note, Atox1-NLS restored wound healing more rapidly than Atox1-WT. These findings indicate that nuclear Atox1 (transcription factor function of Atox1) is required for wound healing process.

### Atox1 is involved in p47phox-ROS-NFkB activation as well as cyclin D1-cell proliferation in wound tissue

**Figure 8:**
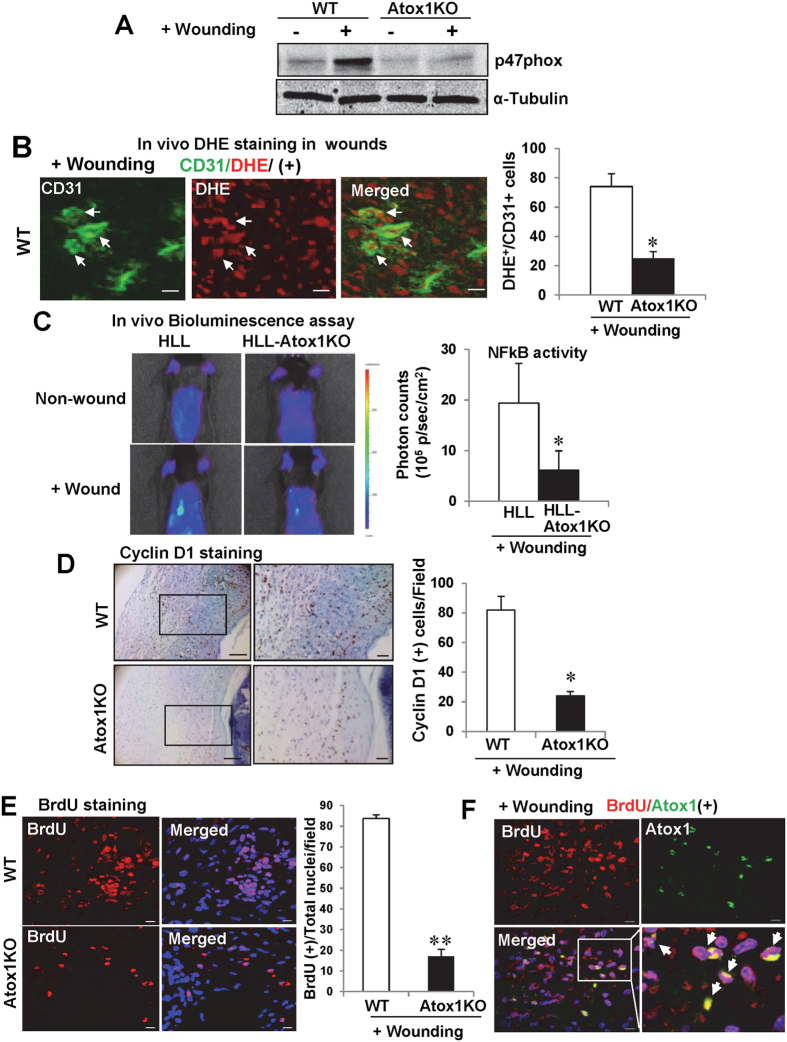
Atox1 is involved in p47phox-ROS-NFkB activation as well as cyclin D1-cell proliferation in wound tissue. (**A**) p47phox and α-tubulin (loading control) protein expression in wound tissues at day 7 in WT and Atox1^−/−^ mice. (**B**) Representative images for CD31 staining (green), DHE fluorescence (red), and their merged images (yellow) in wound tissues at day 7 after wounding in WT mice. DHE+/CD31+ ECs (yellow) are shown in white arrows. Right panel shows mean ± SE of CD31+/DHE+ cells in WT and Atox1^−/−^ mice (n = 3, *p < 0.05 vs. WT). Scale bars = 10 μm (**C**) Representative bioluminescence images of back skin of NFkB activity reporter mice (HLL mice) and HLL/Atox1 KO mice before and at 7 day after wounding (n = 3)(left) and a graph representing mean ± SE of bioluminescence intensity (n = 3, *p < 0.05 vs. HLL). (**D**,**E**) Cell proliferation in wounds were assessed by Cyclin D1 staining, Scale bar = 50 μm (left images); scale bar = 10 μm (right images). (**D**) and BrdU with DAPI (blue, nuclear marker) staining (**E**) at day 5 after wounding in WT and Atox1^−/−^ mice. Graphs represents mean ± SE of cyclin D1^+^ cells and BrdU^+^ cells (n = 3, *p < 0.05; **p < 0.01 vs WT) (**F**) Immunofluorescence showing co-localization of BrdU^+^ cells (red) and Atox1 (Green) in the nucleus in dermal region at day 5 after wounding in WT mice. Scale bars = 10 μm.

We previously reported that Atox1 functions as a Cu-dependent transcription factor for NADPH oxidase organizer p47phox in ECs^25^ as well as cyclin D1 in mouse fibroblasts^24^. This is further supported by findings that nuclear-targeted Atox1 increases cyclin D1 and p47phox expression in ECs (supplemental Fig. 1). Consistently, wound-induced increase in p47phox expression (Fig. 8A) and O_2_^−^ production in ECs (DHE^+^/CD31^+^ cells)(Fig. 8B) in wound tissues were significantly inhibited in Atox1^−/−^ mice at day7 after wounding when contribution of inflammatory cells is minor. Moreover, *in vivo* Bioluminescence imaging of NF-kB reporter (HLL) mice showed that wound injury-induced increase in redox-sensitive NF-kB activity was markedly decreased in HLL mice crossed with Atox1^−/−^ mice (Fig. 8C). Moreover, we found that cyclin D1 + cells (Fig. 8D) as well as BrdU^+^ cells (Fig. 8E), which partially colocalized with Atox1^+^ cells (Fig. 8F) in the nucleus within dermis region in wound tissues, were significantly decreased in Atox1^−/−^ mice. These findings suggest that Atox1 is involved in ROS production and its downstream NF-kB activity via upregulating p47phox as well as cell proliferation via upregulating cyclin D1, thereby promoting wound healing.

### Atox1 is required for Cu enzyme LOX activation and ECM maturation

**Figure 9:**
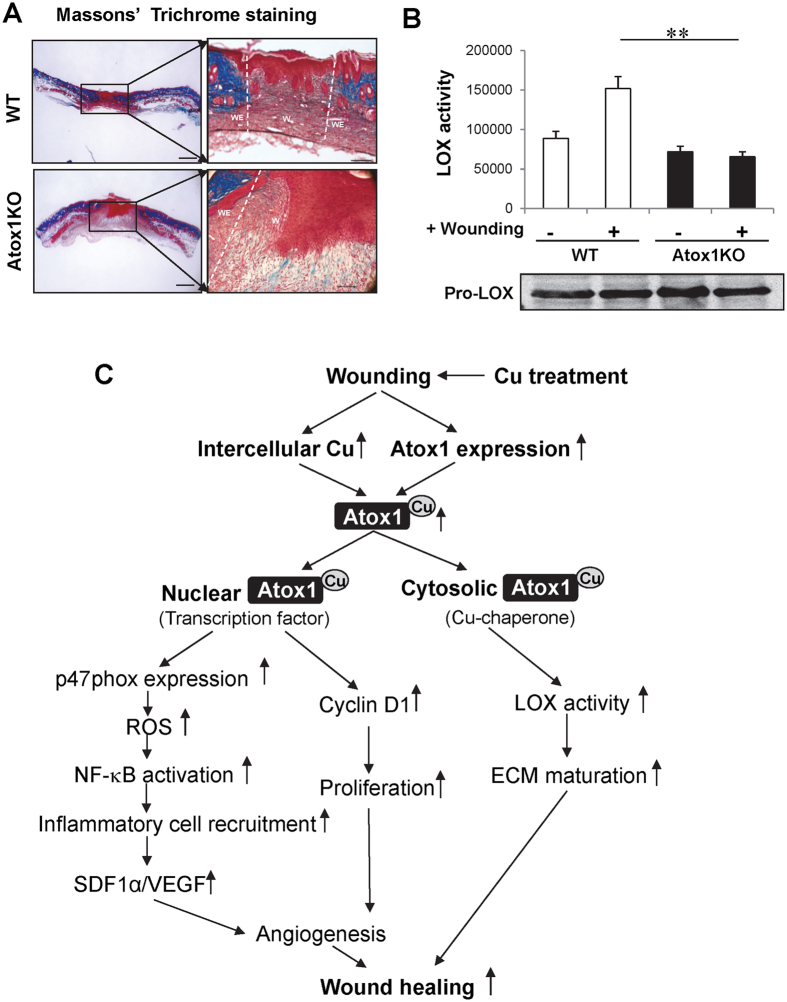
Atox1 is required for ECM maturation and Cu enzyme LOX activation. (**A**,**B**) Masson’s Trichrome staining, scale bars = 500 μm (**A**) and LOX activity (**B**) in wound tissues at day 7 after wounding in WT and Atox1^−/−^ mice. In (**A**) boxed regions are shown at higher magnification to the right, scale bars = 100 μm. Blue color indicates the collagen deposition; light red or pink for keratin, muscle or cytoplasm; and dark brown or black for cell nuclei. W: wound area; WE: wound edge In (**B**) a graph represents mean ± SE for LOX activity and a western blot represents Pro-LOX protein expression in wound tissues at days 0 and 7 (n = 3. **p < 0.01 vs. WT). (**C**) Schematic diagram showing the essential role of Cu-dependent transcription factor and Cu chaperone function of Atox1 in Cu-dependent wound healing.

Masson’s Trichrome staining in Fig. 9A revealed that ECM maturation via collagen deposition was markedly reduced in the healing wound after 7 days in Atox1^−/−^ mice as compared to WT mice. There appears to be an increase in the amount of muscle tissue or keratin presumably due to compensatory response to impaired ECM maturation and angiogenic responses in Atox1^−/−^ wound tissues. Since cytosolic Atox1 functions as a Cu chaperone for secretory ECM Cu enzyme LOX which is involved in ECM maturation during wound healing^19^,^20^,^22^ and VEGF-induced angiogenesis in ECs^25^, we next examined a role of Atox1 for LOX activity in wound tissues. Figure 9B showed that wound injury-induced LOX activity was almost completely abolished in Atox1^−/−^ mice without affecting precursor pro-LOX protein expression. These results suggest that Cu chaperone function of Atox1 is required for ECM maturation by activating LOX which catalyses the cross links and acts collagen deposition or fibrosis during wound healing.

## Discussion

Although the direct role of Cu in promoting angiogenesis and wound healing has been appreciated for several decades^1^,^2^,^3^,^4^,^5^,^6^,^7^,^8^,^9^,^10^,^11^,^12^,^13^, the specific targets of Cu action and underlying mechanisms remain unclear. Using skin puncture wound healing model which is dependent on angiogenesis, inflammation and ECM maturation^15^,^29^,^30^ in global and EC-specific Atox1^−/−^ mice, gene transfer of nuclear-target Atox1 in Atox1^−/−^ mice, and mice treated with Cu chelator, here we demonstrate that Cu-dependent transcription factor Atox1 in ECs plays an important role in wound healing. Mechanistically, angiogenesis, cell proliferation, ROS production, NF-kB-dependent inflammatory response as well as Cu-dependent ECM secretory enzyme LOX activity induced by wound injury are markedly inhibited in Atox1^−/−^ mice. Of note, expression of Atox1 target genes such as p47phox NADPH oxidase and cyclin D1 is also dramatically decreased in wound tissues of Atox1^−/−^ mice. Together with findings that Cu chaperone function of Atox1 regulates LOX activity, our findings should provide insights into how exogenous and endogenous Cu accelerates wound healing via Cu-dependent transcription factor and Cu chaperone function of Atox1 by promoting angiogenesis, inflammatory cell recruitment and ECM maturation (Fig. 9C).

Role of exogenous Cu treatment in enhancing angiogenesis and wound healing has been reported^5^,^6^,^7^,^9^,^10^,^11^,^12^,^13^; however, whether endogenous Cu is required for wound repair remains unknown. In this study, we show that topical treatment of the wounded site with Cu significantly accelerates wound contraction and closures, while Cu chelator BCS impairs the healing process, indicating that endogenous Cu is required for wound healing. We also provide the first evidence that wound injury selectively increases expression of Atox1, but not other Cu chaperones such as CCS or Cox 17, which is associated with increased Cu level in wounding tissues. Functional significance of increased Atox1 in the wounded tissue is demonstrated that Cu treatment had no effect on wound closure in Atox1^−/−^ mice, while Atox1^−/−^ mice showed delayed wound healing. Of note, Cu levels in the non-wounded and wounded tissue in Atox1^−/−^ mice is increased as compared to WT mice. Given that Atox1 is important for Cu exporter ATP7A function, the increased Cu levels in Atox1^−/−^ mice would be partially due to impaired ATP7A function^27^,^35^. This result also indicates that impaired wound healing in Atox1^−/−^ mice is not due to the decreased tissue Cu level, and that increased Cu in Atox1^−/−^ mice is not bioavailable to promote wound healing. Consistent results are previously observed in Atox1^−/−^ aorta in angiotensin II-induced hypertension^32^. Taken together, these findings suggest that Atox1 senses exogenous Cu or endogenously-elevated Cu after wound injury to accelerate wound healing and that the Cu-Atox1 pathway is required for cutaneous wound healing process.

Cu has long been recognized as a key regulator for angiogenesis which plays a critical role in wound healing; however, underlying mechanisms remain unclear^1^,^2^,^3^,^4^,^5^,^6^,^7^,^8^,^9^,^10^,^11^,^12^,^13^. The present study using EC-Atox1^−/−^ mice reveals that Atox1 in EC contributes to wound healing and tissue repair. Furthermore, wounding-induced increase in CD31^+^ ECs (angiogenesis) which express Atox1 is significantly reduced in Atox1^−/−^ mice, thereby inhibiting perfusion recovery in wound tissues. This Atox1-mediated wound angiogenesis might be through several mechanisms. First, we found that Cu treatment markedly increases Atox1 and VEGF expression in wound tissues, which are abolished in Atox1^−/−^ mice. Consistently, previous study shows that Cu addition increases VEGF expression in part via activation of HIF-1α^5^,^36^,^37^. We reported that Atox1 functions as a Cu-dependent transcription factor^24^ and found that HIF1α promoter has Atox1 responsive elements (GAAAGA), which raises the possibility that the Cu-Atox1-HIF1α pathway may upregulate VEGF expression, thereby promoting wound angiogenesis. Second, Atox1 is originally appreciated as a cytosolic Cu chaperone to transfer Cu to the secretory Cu enzymes such as LOX and ecSOD via Cu transporter ATP7A^18^. Thus, the proposed role of Cu in wound healing includes activation of LOX which contributes to maturation of ECM such as collagen and elastin via promoting the cross-linking of the lysine-derived aldehyde^19^,^20^,^21^,^22^. In the present study, we found that Atox1^−/−^ mice exhibit reduced LOX specific activity and collagen deposition in wounded skin compared with WT. This is consistent with our recent report that Atox1 functions as a Cu chaperone to deliver Cu to the secretory Cu enzyme LOX which mediates VEGF-induced angiogenesis in ECs^25^. Thus, these results suggest that Atox1 is involved in Cu-dependent wound healing via promoting ECM maturation and angiogenesis by regulating VEGF expression and LOX activity *in vivo.* We cannot exclude the possibility that Cu chaperone Atox1 also delivers Cu to the ecSOD which plays an important role in ischemia- and wounding-induced angiogenesis, thereby promoting wound healing^38^,^39^,^40^.

In this study, we found that Atox1 is also expressed in Mac3^+^ macrophage infiltrated into wounded sites. It is shown that recruited inflammatory cells following tissue injury secrete angiogenic cytokines and chemokines such as VEGF and SDF1α at the injured sites, thereby promoting angiogenesis and tissue repair^31^,^32^,^33^. We found that Atox1^−/−^ mice show reduced recruitment of monocytes/macrophages, which may contribute to decrease in VEGF and SDF1α expression in wound tissues, leading to impaired angiogenesis and healing. Thus, it is possible that Atox1-dependent upregulation of VEGF protein in wound tissues may be in part mediated through activation of the Cu-Atox1-HIF1α pathway in inflammatory cells. Thus, we cannot exclude the involvement of Atox1 in macrophages in Atox1-dependent wound healing. This may explain why impaired wound healing in EC-specific Atox1 KO mice seems less severe than the impairment seen in global Atox1 KO mice. Of note, re-epithelialization after wound injury in Atox1^−/−^ mice is increased, as compared to control mice (Fig. 2A), which may be partly due to compensatory response to impaired ECM maturation and angiogenic response. Re-epithelialization which involves keratinocyte^41^ largely contributes to human skin wound healing, but not in mouse skin puncture model. Thus, role of Atox1 in re-epithelization is not clear in this study and requires detailed analysis using mouse wound splint model^42^, or keratinocyte specific Atox1 deficient mice.

As described above, we reported that Atox1 also functions as a Cu-dependent transcription factor^24^; however, the role of nuclear Atox1 *in vivo* remains unknown. In this study, immunofluorescence and subcellular fractionation analysis reveal that nuclear Atox1 expression is increased in some dermal CD31^+^ ECs after wounding. Moreover, gene transfer of nuclear-targeted Atox1 in Atox1^−/−^ mice rescues impaired wound healing, suggesting an important role of nuclear Atox1 in this response. Mechanistically, wounding-induced p47phox expression as well as O_2_^−^ production in CD31^+^ ECs detected by DHE are markedly inhibited in Atox1^−/−^ mice. Of note, it is reported that p47phox-derived H_2_O_2_ facilitates wound closure *in vivo*^42^. Furthermore, *in vivo* Bioluminescence imaging of HLL mice demonstrates that wounding-induced NF-kB activity is significantly inhibited in Atox1^−/−^ mice, which is associated with a decrease in expression of its downstream target adhesion molecule VCAM1 expression in wound tissues. Consistent with current study using cutaneous wound healing model, we reported that Atox1 translocates to nucleus in response to proinflammatory cytokine in ECs and functions as a Cu-dependent transcription factor for p47phox to increase the ROS-NF-kB pathway and adhesion molecule expression in inflamed cultured ECs^25^. Thus, it is conceivable that reduced inflammatory cell recruitment in Atox1^−/−^ mice may be secondary due to the attenuation of ROS-NFkB-VCAM1 pathway in ECs, which may also contribute to decrease in p47phox and ROS production in wounded tissue. Moreover, the present study also shows that wounding-induced cyclin D1^+^ cells within dermis region and cell proliferation (BrdU^+^ cells) are markedly decreased in wound tissues of Atox1^−/−^ mice. This is consistent with our previous report that nuclear Atox1 stimulates Cu-dependent cell proliferation through Cyclin D1 in mouse fibroblast cells^24^. Thus, these results suggest that nuclear transcription factor function of Atox1 plays an important role in wound healing and repair process by promoting reparative inflammatory cell recruitment and cell proliferation at least via increasing transcription of p47phox and cyclin D1. Other targets of Atox1 involved in wound healing should be clarified in future study.

In conclusion, our study suggests that endothelial Atox1 senses Cu to accelerate wound healing/repair by promoting ROS production, inflammatory cell recruitment and cell proliferation via Cu-dependent transcription factor for p47phox and cyclin D1 as well as ECM maturation via Cu chaperone function for secretory Cu enzyme LOX. (Fig. 9C) Thus, enhancing the Cu-Atox1-mediated therapy may represent a novel therapeutic approach to promote dermal wound healing which is dependent on angiogenesis and inflammation.

## Methods

### Animal

Atox1^−/−^ mice (backcrossed eight times to C57Bl/6) were obtained from Mutant Mouse Regional Resource Centers. They were further backcrossed to C57Bl/6 mice more than ten times, as previously described^27^. Age matched C57Bl/6 mice used for wild type (WT) mice were purchased from Jackson Laboratory. All mice were maintained at the University of Illinois at Chicago animal facilities. Mice at 12 to 16 weeks-old were used for experiments. All studies were carried out in accordance with the guidelines approved by the Animal Care and Use Committee of the University of Illinois-Chicago.

To generate EC-specific Atox1 KO mice, we first established Atox1 floxed mouse line using Atox1 floxed ES cells obtained from European Conditional Mouse Mutagenesis Program (EUCOMM). We removed NEO resistance gene by breeding with Flp recombinase expressing transgenic mouse. EC-specific Atox1 KO mice were generated by crossing Atox1 floxed mice (Atox1 fl/fl) with VEcad-Cre+/− transgenic mouse (Jackson Laboratory).

### Full thickness excisional wounds

Twelve weeks old Atox1^−/−^ and C57BL/6 mice were used for cutaneous wound healing experiments. To compare the wound closure rates between Atox1^−/−^ and C57Bl/6 mice, full-thickness 3-mm skin punch biopsy (Acuderm) was created on the dorsal skin, as previously described^44^. For healing kinetic analysis, digital images of wounds were taken and wound diameters were measured using Image J software. For drug treatment, a larger 6-mm skin punch biopsy was used to add 25 μl of reagents on wounds. Wounds were topically treated with either 25 μl of 50 μM CuCl_2_ (1.25 nmoles by weight), Cu-chelator BCS (400 μM, 10.01 nmoles by weight), or vehicle (PBS). Wounds were dressed with bioclusive transparent oxygen-permeable wound dressing (Johnson & Johnson) and changed every two days.

### Immunoblotting

Wound tissues were homogenized in RIPA buffer (5 mM Tris-HCl (pH 7.6), 150 mM NaCl, 1% NP-40, 1% sodium deoxycholate, 0.1% SDS) with protease inhibitor followed by brief sonication as described previously^25^. Equal amount of protein was separated by SDS-PAGE. Following primary antibodies were used: anti-Atox1^24^, anti-p47phox (Millipore), anti-VEGF (Neo Markers), anti-SDF1α (Cell Sciences) or VCAM1 antibody (Santa Cruz). Protein expression was visualized by ECL (Amersham). Band density was quantified by ImageJ. Nuclear/cytoplasmic fractionation of skin tissue was performed using an NEPER Nuclear and Cytoplasmic Extraction Reagents Kit (Pierce) according to manufacturer’s protocol as described previously^24^,^27^.

### Quantitative real-time PCR

Total RNA of skin was isolated by using phenol/chloroform and isolated using Tri Reagent (Molecular Research Center Inc.). Reverse transcription was carried out using high capacity cDNA reverse transcription kit (Applied biosystems) using 2 ug of total RNA. Quantitative PCR was performed with the ABI Prism 7000, the SYBR Green PCR kit (Qiagen) and the QuantiTect Primer Assay (Qiagen) for specific genes. Samples were all run in triplicates to reduce variability. Expression of genes was normalized and expressed as fold-changes relative to GAPDH.

### Copper Measurements by Inductively coupled plasma mass spectrometry (ICP-MS)

Samples were diluted to 2.0 ml with deionized water containing 5% v/v nitric acid. The copper contents were analyzed by ICP-MS, using a PlasmaQuad3, as previously described^27^,^45^. Copper concentrations were calculated from calibration curves, and values for water blank were subtracted.

### Synchrotron X-ray Fluorescence Microscopy

Sections (5 μm thick) of formalin-fixed paraffin-embedded skin tissue were used. For X-ray imaging, the sections were mounted intact on silicon nitride windows (area, 2 × 2 mm; thickness, 200 nm) manufactured by Silson (Blisworth, U.K.) and attached by brief heating to 55 °C, as previously described^27^,^45^. Specimens were imaged with the scanning X-ray fluorescence microprobe at beamline 2-ID-E of the Advanced Photon Source (Argonne, IL). Undulator-generated x-rays of 10-keV incident energy were monochromatized with a single bounce Si monochromator and focused to a measured spot size of 0.3 × 0.5 μm using Fresnel zone plate optics (X-radia, Concord, CA). Sections were raster-scanned in steps of 4.0 μm, and fluorescence spectra were collected for 1- to 2-sec dwell times by using a single-element silicon drift detector (Vortex-EX, SII Nanotechnology, CA). Quantitation and image-processing of the X-ray fluorescence (XRF) data sets was performed with MAPS software. Quantitation of elemental content was achieved by fitting XRF spectra at each pixel, and comparing against a calibration curve derived from measurements of thin-film standards NBS-1832 and NBS-1833 (National Bureau of Standards, Gaithersburg, MD).

### Histology and immunohistochemistry

Mice were sacrificed at 1–7 days after wounding, and skin tissues around wound edge were harvested and embedded in paraffin. Frozen sections were prepared by overnight 4% PFA incubation followed by sucrose dehydration and OCT embedding. 5-μmthick sections were stained with hematoxylin-eosin (HE), and Masson’s trichrome staining. To determine the Capillary density, wound tissue sections were stained with anti-mouse CD31 antibody (BD Biosciences) followed by biotinylated anti-mouse IgG antibody (Vector Laboratories). For other immunohistochemical analysis, skin sections were incubated with primary antibodies against Atox1, Mac-3 (BD Biosciences) and cyclin D1 (Abcam). This was followed by incubation with biotin-conjugated secondary antibody (Vector Laboratories) Next, we used R.T.U. Vectorstain Elite (Vector Laboratories) followed by DAB visualization (Vector Laboratories). Immunofluorescence staining was performed with primary antibodies against Atox1, CD31 (BD Biosciences), or Mac-3 (BD Biosciences). Secondary antibodies were Alexa Fluor 488 or 546-conjugated goat anti-rabbit IgG and goat anti mouse IgG (Invitrogen). In each experiment, TO-PRO-3 (Invitrogen) was used for nuclear counter staining. Images were captured by Axio scope microscope (Zeiss) or confocal microscopy (Zeiss) and processed by AxioVision 4.8 or LSM510 software (Zeiss).

### Blood flow imaging

We measured blood flow in skin wound edge area using a laser Doppler blood flow (LDBF) analyzer (PeriScan PIM 3 System; Perimed) as previously described^25^,^43^. Mice were anesthetized and placed on a heating plate at 37 °C for 10 minutes to minimize temperature variation. Before and after wounding, LDBF analysis was performed in the plantar sole. Blood flow was displayed as changes in the laser frequency, represented by different color pixels.

### Plasmids, deletions, site-directed mutagenesis

For nuclear targeted Atox1 (Flag-Atox1-NLS) constructs, a tripartite NLS sequence (PKKKRKVD) derived from SV40 large T antigen was fused to the the C-terminus of Flag tagged WT-Atox1, as we previously described^24^. For creation of lentivirus, these Atox1 mutants (Flag-Atox1-WT, Flag-Atox1-NLSSV^41^) were subcloned into the pLL3.7 lentivirus shuttle vector with a CMV promoter-MCS-IRES-GFP^46^,^47^.

### Generation of Lentivirus and *in vivo* treatment

Lentivirus was produced by a triple transfection of the pLL3.7 lentivirus shuttle vector with control or various Atox1 mutants, pΔ8.9, and pVSVG as described^46^. In brief, pLL3.7 and packaging vectors were cotransfected into HEK293FT cells using Lipofectamine 2000 (Invitrogen); supernatant was collected after 48 h and passed thorough a 0.45-μm filter to remove debris. 293FT-conditioned media were filtered and further ultracentrifuged by ultrafiltration (Amicon Ultra 100,000 MW cutoff; Millipore Corporation) to concentrate the vector. An approximate viral titer was determined by infecting HEK293 cells with serial dilutions of the final virus suspension and counting the number of fluorescent cells 48 h after infection. For infection of lentivirus expressing either control GFP vector virus or various Atox1 expressing virus (CMV-Atox1-WT-IRES-GFP, or CMV-Atox1-NLS-IRES-GFP) in wounds, 1 × 10^6^ transduction units/ml of lentivirus were treated in Atox1^−/−^ mice, as described above.

### O_2_^•−^ Detection in Mice

Dihydroethidium (DHE) (Invitrogen, Grand Island, NY) was prepared as a 1 mg/ml solution in 1% Dimethyl sulfoxide (DMSO) and administered at 10 mg per kg of body weight by intravenous injection as described previously^25^,^31^. Mice were killed and immediately perfusion-fixed with 4% paraformaldehyde 1 hour after DHE injections. Frozen sections of skin were prepared and observed by confocal microscopy (Zeiss, Thornwood, NY) with excitation at 510–550 nm and emission >585 dsx7Anm to detect oxidized ethidium.

### *In vivo* bioluminescence assay

HLL and Atox1KO-HLL mice were anesthetized, and the hair was removed over the back side of mice before imaging as described previously^25^. Luciferin (150 mg/kg/mouse in 200 μl of isotonic saline) was administered by i.p. injection. After 12–16 min, mice were imaged using Xenogen IVIS spectrum (Caliper Life Sciences, Hopkinton, MA, USA). For the duration of photon counting, mice were placed inside a light-tight box. Light emission from the mouse was detected as photon counts using the intensified charge-coupled device camera and expressed as photon counts. Images were obtained before and following skin wound puncture.

### BrdU staining *in vivo*

To quantify proliferating cells, mice were injected intraperitoneally with the thymidine analog 5-bromo-2′-deoxyuridine (BrdU; 40 mg/kg body weight, 500 μl, SigmaAldrich) at 12 hours and 1 hour before sacrifice as described previously^48^. Mice were euthanized and perfused through the left ventricle with saline, and skin were embedded in OCT compound and snap-frozen in liquid nitrogen. Sections were incubated with a rat anti-BrdU antibody (Abcam) to detect proliferating cells. For nuclear staining, BrdU-labeled tissue sections were counterstained with TO-PRO-3 (Invitrogen).

### LOX activity assay

LOX activity tissue lysate was measured by a high-sensitivity fluorescence assay as previously described^23^. Skin tissues were homogenate in 1X LOX Urea buffer and estimated protein in standard method. Equal amount of protein samples was incubated in the presence and absence of 500 μmol/L BAPN at 37 °C for 30 min with final reaction mixture supplied by Amplite Fluorimetric Lysyl Oxidase Assay kit (AAT Bioquest) to the manufacturer’s instruction. The reaction was stopped on ice, and differences in fluorescence intensity (540-nm excitation wavelength and 590-nm emission wavelength) between samples with and without BAPN were determined. Specific activity was determined by the ratio of activity to relative amount of protein.

### Statistical analysis

Results are expressed as means ± SE. Statistical significance was assessed by Student’s paired two-tailed *t*-test or ANOVA on untransformed data, followed by comparison of group averages by contrast analysis using the Super ANOVA statistical program (Abacus Concepts, Berkeley, CA). A p value of ±0.05 was considered to be statistically significant.

## Additional Information

**How to cite this article**: Das, A. *et al*. Endothelial Antioxidant-1: a Key Mediator of Copper-dependent Wound Healing *in vivo*. *Sci. Rep.* **6**, 33783; doi: 10.1038/srep33783 (2016).

## Supplementary Material

Supplementary Information
