# Supplementary material for: Endothelial Antioxidant-1: a Key Mediator of Copper-dependent Wound Healing in vivo
Source: Sci Rep. 2016 Sep 26;6:33783. doi: 10.1038/srep33783 (PMC5036036; doi:10.1038/srep33783)
Supplement: Supplementary Information [file srep33783-s1.pdf]

SUPPLEMENTARY INFORMATION

Endothelial Antioxidant-1: a Key Mediator of Copper-dependent Wound Healing *in vivo*

Archita Das[1], Sudhahar Varadarajan[1,2], Gin-Fu Chen[1], Ha Won Kim[1], Seock-Won Youn[3], Lydia Finney[4], Stefan Vogt[4], Jay Yang[5], Junghun Kweon[1,2+], Bayasgalan Surenkhuu[1,2], Masuko Ushio-Fukai[,3], Tohru Fukai[1,2]

From [1]Departments of Medicine (Section of Cardiology) and Pharmacology, Center for Cardiovascular Research, Center for Lung and Vascular Biology, University of Illinois at Chicago, Chicago, IL60612

[2]Jesse Brown Veterans Affairs Medical Center, Chicago, IL60612

[3]Department of Pharmacology, Center for Lung and Vascular Biology, Center for Cardiovascular Research, University of Illinois at Chicago, Chicago, IL60612

[4]Argone National Laboratory, IL60439

[5]Department of Anesthesiology, University of Wisconsin, Madison, WI 53792

Short running title: Endothelial Atox1 and wound healing

Address correspondence to:

Tohru Fukai, M.D., Ph.D.
Depts. of Medicine (Section of Cardiology) and Pharmacology,
Center for Cardiovascular Research,
University of Illinois at Chicago,
835 S. Wolcott, M/C868
E403 MSB
Chicago, IL60612
Phone: 312-996-7631 (office)
Fax: 312-996-1225
Email: tfukai@uic.edu

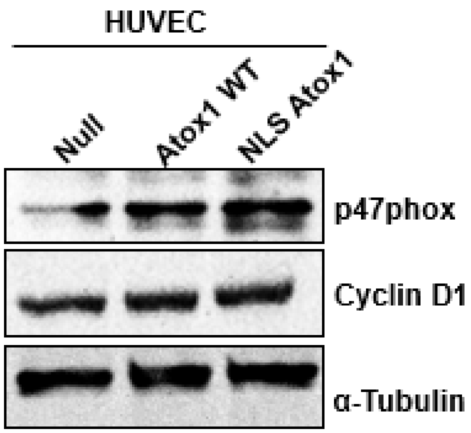

Supplemental Figure 1. Nuclear-targeted Atox1 increases expression of Atox1 target proteins (p47phox and cyclin D1) in ECs. HUVECs infected with adenovirus expressing Atox1-WT or Atox1 with nuclear-target sequence (Atox1-NLS) were used to measure p47phox, cyclin D1, or α-tubulin (loading control) protein expression using western analysis.
